# Multilevel interaction of the DnaK/DnaJ(HSP70/HSP40) stress-responsive chaperone machine with the central metabolism

**DOI:** 10.1038/srep41341

**Published:** 2017-01-27

**Authors:** Fréderic Anglès, Marie-Pierre Castanié-Cornet, Nawel Slama, Mickael Dinclaux, Anne-Marie Cirinesi, Jean-Charles Portais, Fabien Létisse, Pierre Genevaux

**Affiliations:** 1 Laboratoire de Microbiologie et de Génétique Moléculaires, Centre de Biologie Intégrative (CBI), Université de Toulouse, CNRS, 118 route de Narbonne, 31062 Toulouse Cedex 9, France; 2 LISBP, Université de Toulouse, CNRS, INRA, INSA F-31400 Toulouse, France

## Abstract

Networks of molecular chaperones maintain cellular protein homeostasis by acting at nearly every step in the biogenesis of proteins and protein complexes. Herein, we demonstrate that the major chaperone DnaK/HSP70 of the model bacterium *Escherichia coli* is critical for the proper functioning of the central metabolism and for the cellular response to carbon nutrition changes, either directly or indirectly via the control of the heat-shock response. We identified carbon sources whose utilization was positively or negatively affected by DnaK and isolated several central metabolism genes (among other genes identified in this work) that compensate for the lack of DnaK and/or DnaK/Trigger Factor chaperone functions *in vivo*. Using carbon sources with specific entry points coupled to NMR analyses of real-time carbon assimilation, metabolic coproducts production and flux rearrangements, we demonstrate that DnaK significantly impacts the hierarchical order of carbon sources utilization, the excretion of main coproducts and the distribution of metabolic fluxes, thus revealing a multilevel interaction of DnaK with the central metabolism.

Ubiquitous molecular chaperone machines control cellular protein homeostasis, both under lenient and more stressful growth conditions^1^. Among these machines, members of the highly conserved HSP70 (Heat Shock Protein of 70 kDa) family of ATP-dependent molecular chaperones have been associated with almost all known chaperone functions^2^,^3^. In the bacterium *Escherichia coli*, the multifunctional HSP70, named DnaK, acts in concert with its cochaperones DnaJ and GrpE (all together referred as DnaKJE) to efficiently assist *de novo* protein folding, protein disaggregation, protein targeting and translocation through biological membranes, as well as the remodeling of protein complexes^4^,^5^,^6^,^7^. DnaK interacts with a wide range of *E. coli* proteins, more than 700^8^ and mutations in *dnaK* induces a plethora of cellular defects that result in various phenotypes such as a cold- and a temperature-sensitive growth, a susceptibility to nutrient starvation and to various antibiotics^3^,^9^.

It is known that DnaKJE downregulates the entire heat shock response (HSR) by interacting directly with heat shock transcription factor σ^32^ to facilitate its degradation by the membrane-anchored FtsH protease^10^. Under stress condition, DnaK is efficiently recruited to accumulating aggregated proteins, leading to an increased stability of σ^32^ and the subsequent induction of more than hundred HSPs^11^. This places DnaK as a central component of the cellular response to proteostasis collapse, both by acting directly on misfolded and aggregated proteins and by modulating the synthesis of a plethora of other heat shock inducible chaperones and proteases.

Remarkably, previous works showed that DnaKJE acts in a concerted and cooperative manner with other major cytosolic chaperones, namely Trigger Factor (TF) and GroESL, especially during the biogenesis of cytosolic proteins^8^,^12^. Although the ribosome-bound TF facilitates the folding of the majority of the newly synthesized *E. coli* proteins, a significant fraction of these proteins, over 30%, necessitates assistance by the downstream DnaKJE and GroESL chaperones to complete their folding. Deletion of the *tig* gene encoding TF enables DnaK to interact with shorter nascent chains and increases the number of DnaK interactors by more than 35%, indicating that substrates of both chaperones overlap significantly. In addition, TF and DnaK can sequentially interact with large multi-domain proteins and actively cooperate to promote their folding^13^. Accordingly, simultaneous deletion of both the *tig* and *dnaK* genes is synthetic lethal at temperatures ≥30 °C and induces a strong accumulation of cytosolic protein aggregates containing more than one thousand different proteins, both pre-existing and newly synthesized^8^,^14^,^15^,^16^.

The recently described *in vivo* interactome of DnaK in *E. coli* revealed that at least 50% of the central metabolism (CM) enzymes interact with DnaK^8^. Previous work also showed that *dnaK* mutants cannot grow on mannose or sorbitol as sole carbon sources^17^, and that DnaK expression is subject to a strong Crp-mediated catabolite repression^18^. Taken together, these results suggest an important, yet unexplored role of DnaK in both the proper functioning of the CM and the metabolic shifts in response to carbon nutrition changes. Remarkably, through a multicopy suppression analysis we have identified six genes of the central metabolism (CM), namely *ackA, ldhA, lpd, pykF, talB* and *csrC,* that partially suppress the growth defect of the sensitive Δ*tig* Δ*dnaKJ* chaperone mutant, with the three first also suppressing the growth defect of the single Δ*dnaKJ* mutation at high temperature, thus strongly suggesting a major role of DnaK in this process. Using a combination of growth assays on carbon sources with specific entry points in the CM coupled to a functional analysis of the metabolism, we next demonstrate that DnaK significantly impacts the responsiveness of the CM by acting either directly at the level of the CM or along the first steps of carbon assimilation. How does the multifunctional DnaK chaperone modulate carbon consumption in response to proteostasis failure or in response to nutrient starvation is discussed.

## Results

### Central metabolism genes support bacterial growth in the absence of major chaperones

**Figure 1:**
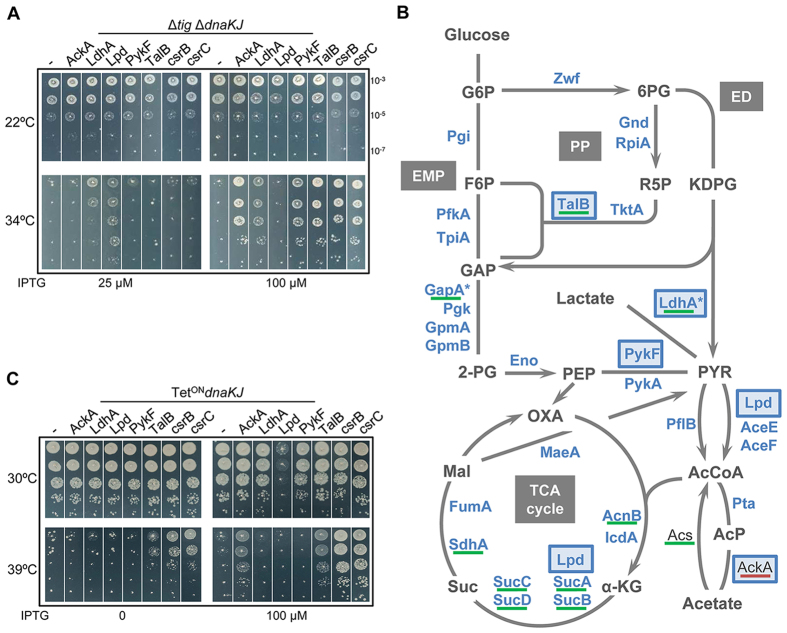
Central metabolism genes rescue bacterial growth in the absence of major chaperones. (**A**) MG1655 Δ*tig* Δ*dnaKJ* mutant containing plasmid pSE380NcoI vector (−), pSE-AckA, pSE-LdhA, pSE-Lpd, pSE-PykF, pSE-TalB, pSE-csrB or pSE-csrC were grown at 22 °C, serially diluted 10-fold, and spotted on LB ampicillin agar plates with (25 or 100 μM) or without IPTG inducer. Plates were incubated for 1 day at 34 °C or 2 days at 22 °C. (**B**) Schematic representation of previously identified *in vivo* DnaK interactors by Calloni and coworkers (2012) within the central metabolic network. Interactors of DnaK are depicted in blue and newly identified suppressors from (A) are highlighted with a dark blue frame. Proteins significantly increased in the Δ*dnaK* mutant are underlined in green and those significantly decreased in red. Heat-shock proteins are marked with an asterisk. Metabolic network includes the Embden–Meyerhof–Parnas (EMP) pathway, the Pentose Phosphate (PP) pathway, the Entner-Doudoroff (ED) pathway and the Tricarboxylic Acid cycle (TCA cycle). Abbreviations: glucose-6-phosphate (G6P), fructose-6-phosphate (F6P), 6- phosphogluconate (6PG), ribose-5-phosphate (R5P), 2-keto-3-deoxy-6-phospho-gluconate (KDPG), glyceraldehyde-3-phosphate (GAP), 2-phospho-D-glycerate (2-PG), phosphoenolpyruvate (PEP), pyruvate (PYR), acetyl-CoA (AcCoA), acetyl-phosphate (AcP), α-ketoglutarate (α-KG), succinate (Suc), malate (Mal) and oxaloacetate (OXA). (**C**) Transformants of MG1655 PTet^ON^ *dnaKJ* containing plasmid pSE380NcoI vector, pSE-AckA, pSE-LdhA, pSE-Lpd, pSE-PykF, pSE-TalB, pSE-csrB or pSE-csrC were grown to mid-log phase at 30 °C in LB supplemented with ampicillin and anhydrotetracycline to ensure expression of DnaKJ, serially diluted 10-fold, and spotted on LB ampicillin agar plates with or without IPTG. Note that the anhydrotetracycline inducer was not present in the plates to ensure the repression of the *dnaKJ* operon. Plates were incubated for 1 day at 30 °C or at 39 °C.

The Δ*tig* Δ*dnaKJ* chaperone-deficient strain is a sensitive genetic tool to study proteostasis networks in bacteria and identify novel factors involved^15^,^19^,^20^. Our search for overexpressed *E. coli* genes that partially suppress the bacterial growth defect observed in the absence of these main chaperones led to the identification of several new genes involved in multiple cellular processes including transcription (*dksA, csrC, fis*), protein synthesis (*leuX, serX, proL*), tRNA maturation (*rnt*), oxidative stress (*tpx*), metabolism (*ldhA, lpd, ackA, talB, pykF, nagB, sseA, ydfG*) and a gene with unknown cellular functions (*ypaB*). This indicates that cells can use multiple pathways to counteract the severe proteostasis collapse induced by the lack of chaperones, as previously proposed^8^,^15^,^19^,^20^,^21^. A complete list of the newly identified suppressors is shown in Fig. S1. Remarkably, we found that about one third of the multicopy suppressors isolated carried genes that are key players of the CM, which is defined as the main pathways for cell anabolism and catabolism, including the Embden–Meyerhof–Parnas (EMP) pathway, the Pentose Phosphate (PP) pathway, the Entner-Doudoroff (ED) pathway and the Tricarboxylic Acid cycle (TCA cycle). The CM genes include *ackA* encoding the acetate kinase AckA that performs the reversible conversion of acetyl-phosphate (AcP) into acetate, *ldhA* encoding the heat shock protein lactate dehydrogenase LdhA, mainly responsible for the production of D-lactate from pyruvate, *lpd* encoding the lipoamide dehydrogenase Lpd that is part of the glycine cleavage, the pyruvate dehydrogenase and α-ketoglutarate dehydrogenase complexes, *pykF* encoding the pyruvate kinase PykF, which converts phosphoenolpyruvate into pyruvate, *talB* encoding the transladolase B enzyme of the non-oxidative branch of the pentose phosphate pathway, and *csrC*, a small non coding RNA regulating negatively CsrA, a central metabolism regulator^22^. These genes were individually cloned in the plasmid pSE380 under the control of an IPTG-inducible promoter and suppression of the Δ*tig* Δ*dnaKJ* double mutant growth defect at high temperature was confirmed (Fig. 1). In all cases, we found that suppression was optimal at 34 °C and not visible at 37 °C. Note that in addition to *csrC* we have cloned and expressed *csrB*, a non-coding RNA homologous to csrC, also known to negatively regulate CsrA, and found that it equally suppresses the temperature-sensitive (Ts) phenotype (Fig. 1A). This suggests that a shutdown of CsrA regulatory mechanism helps cells to survive in the absence of chaperones^22^.

Partial suppression of the growth defect by genes of the CM suggests that some suppressors could possess chaperone function or be *bone fide* TF/DnaK substrates that do not fold very efficiently (or are degraded) in the absence of the chaperones, and whose overexpression restores a sufficient pool of active proteins. Some of these gene products might also have a high propensity to be unfolded and to aggregate, which could induce another stress response that potentially facilitates growth in the absence of TF and DnaK. In addition, with respect to the nature of the suppressors, their overexpression might also reroute metabolic reactions to bypass the need for TF and DnaK functions. This could occur either through their enzymatic properties or through transcriptional or post-transcriptional remodeling, as it might be the case for csrB/C via a shutdown of CsrA regulatory mechanism^22^, or for AckA via its effect on AcP synthesis (see below). Although suppression by TalB remains enigmatic, the fact that Lpd, PykF and LdhA suppress the growth defect of the Δ*tig* Δ*dnaKJ* double mutant suggests that the TF/DnaK chaperone pathway might contribute at the level of the pyruvate node, which involves the assembly of the large pyruvate dehydrogenase multimeric complex composed of 60 subunits of AceE, AceF and Lpd^23^. In support of this, AceE, AceF and Lpd, were previously isolated as protein aggregates both in the single Δ*dnaK* strain and, more severely, in the double Δ*tig* Δ*dnaK* mutant^8^,^12^.

Interestingly, the recent DnaK interactome performed in *E. coli* wild type revealed that DnaK interacts with a large number of CM enzymes (about 50%, representing 5% of the proteome), including the suppressors LdhA, Lpd, PykF and TalB, and that endogenous expression of at least ten CM enzymes, including TalB and LdhA, increases significantly in the Δ*dnaKJ* mutant (Fig. 1B). Of note, the suppressor AckA was the only CM enzyme exhibiting a lower endogenous level in the absence of DnaKJ^8^. Therefore, we next asked whether the newly isolated multicopy suppressors of the Δ*tig* Δ*dnaKJ* mutant could also suppress the Ts phenotype of DnaK depleted cells^3^. The plasmid-encoded suppressors were overexpressed in the MG1655 P*tet*^ON^*dnaKJ* strain in which the *dnaKJ* operon is repressed in the absence of the anhydrotetracycline inducer^24^ (Fig. S2). The results presented in Fig. 1C show that overexpression of AckA, TalB, csrB or csrC was sufficient to suppress the Ts phenotype of the *dnaKJ* mutant at 39 °C. The fact that some of the isolated CM genes partially suppress the Ts phenotype of the double Δ*tig* Δ*dnaKJ* but not the one of the single *dnaKJ* mutant at high temperature, *i.e*., *pykF, ldhA* and *lpd*, suggests that these genes might be weaker suppressors of the defective DnaK/TF folding pathway, perhaps not capable of supporting folding of critical high-temperature sensitive substrates of both TF and DnaK. Alternatively, suppression might be restricted to TF function, which could be supported by DnaK in its absence. In addition, we found that overexpression of Lpd was toxic at permissive temperature in the *dnaKJ* mutant but had only very little, or no effect in the Δ*tig* Δ*dnaKJ* double mutant (Fig. 1A and C). Further genetic characterization of these suppressors showed that there is no synthetic lethality between a *dnaK* mutation and mutations in the multicopy suppressor genes. Yet, in the case of *lpd*, we found that the double *lpd* Δ*dna*KJ mutant was more severely affected for growth at the normally permissive-temperature of 30 °C when compared to the single isogenic mutants (Fig. S2). Together these data highlight important links between DnaK function and the CM.

### DnaK differentially impacts *E. coli* growth on specific carbon sources

**Figure 2:**
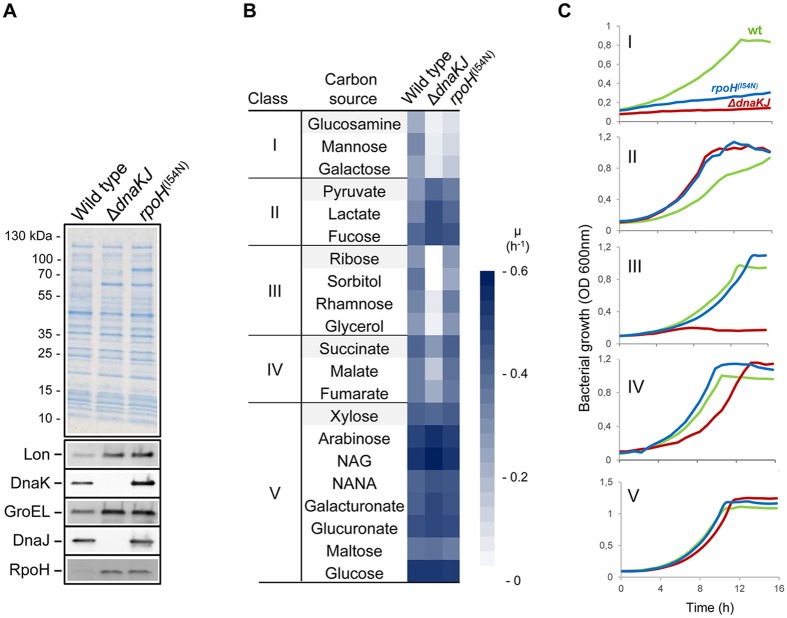
DnaK’s impact on *E. coli* growth is carbon source-dependent. (**A**) Whole cell extracts of MG1655 wild type, Δ*dnaKJ* and *rpoH*^(I54N)^ were separated on SDS-PAGE, stained with Coomassie Blue or analyzed by western blot using anti-Lon, -DnaK, -GroEL, -DnaJ or -RpoH antibodies. (**B**) Heat maps representing the averaged growth rates obtained from three different biological replicates of the three strains grown on 21 carbon sources. The blue scale indicates high (darker) to low (lighter) growth rate. Carbon sources were grouped into five classes each representing a different growth behavior of either the Δ*dnaKJ* or the *rpoH*^(I54N)^ mutant compared to the wild type: class I groups carbon sources on which the two mutants did not grow, class II groups carbon sources on which both mutants exhibit higher growth rates than the wild type, class III groups carbon sources on which a growth defect of the *dnaKJ* mutant is observed, class IV groups carbon sources on which *dnaKJ* mutant exhibits a lowest rate of growth compared to both the wild-type strain and the *rpoH*^(I54N)^ mutant and class V groups carbon sources on which no significant difference in growth was observed for both mutants and the wild type. Abbreviations NAG and NANA stand for N-acetyl-glucosamine and N-acetyl-neuraminate, respectively. **(C**) Representative growth curves of *E. coli* K-12 MG1655 wild type (green), Δ*dnaKJ* (red) and *rpoH*^(I54N)^ (blue) for each class: Glucosamine (class I), Pyruvate (class II), Ribose (class III), Succinate (class IV) and Xylose (class V).

To further investigate such possible interplay between DnaK and the CM, we first tested whether specific patterns of carbon utilization could be observed in the absence of DnaK, depending on the entry point of the carbon sources in the CM. As stated above, one of the key cellular functions of DnaK is the downregulation of the entire HSR. In order to distinguish between an HSR-dependent or -independent role of DnaK in the utilization of specific carbon sources, two mutant derivatives were used throughout this study: a Δ*dnaKJ* mutant, thus lacking all DnaK functions, and a σ^32^ mutant, *i.e*., *rpoH*^(I54N)^, known to escape the DnaK-dependent targeting to FtsH^25^. This σ^32^ mutant harbors the isoleucine 54 to asparagine amino acid substitution in the first part of the second region defined as a small patch with a crucial function in σ^32^ degradation^25^. The I54N mutation almost completely abolishes the DnaK-dependent downregulation of the HSR without appreciably altering σ^32^ binding to RNA polymerase^25^. The use of such a mutant mimics a condition in which DnaK is present and where all the HSPs (including DnaK) are induced to levels comparable to those observed in a *dnaKJ* null mutant. Accordingly, steady state levels of σ^32^ and other HSPs were similar both in MG1655 *rpoH*^(I54N)^ and MG1655Δ*dnaKJ* mutant strains (Fig. 2A).

The three isogenic strains were independently grown at the permissive temperature of 30 °C in minimal medium supplemented with single carbon sources (21 different carbon sources were tested in total) with selected entry points in the CM (Fig. S3), and growth rates were measured for each condition (Fig. 2B and Table S1). From these data, we defined 5 major classes of carbon sources according to the growth behavior of both Δ*dnaKJ* and *rpoH*^(I54N)^ mutants compared to wild type. Class I is represented by carbon sources that do not support growth of both mutants, thus suggesting that the HSR is detrimental in this case; class II carbon sources support higher growth rates for both mutants, which suggests a positive contribution of the HSR; class III carbon sources only support growth of both wild type and *rpoH*^(I54N)^, and class IV contains carbon sources on which the Δ*dnaKJ* mutant exhibits a reduced growth rate when compared to both wild type and *rpoH*^(I54N)^. Both class III and IV thus contain carbon sources for which DnaK likely plays a specific role independently of the HSR. Finally, class V represents carbon sources for which no significant difference could be observed between the strains (Fig. 2B). A representative growth is shown for each class in Fig. 2C. Growth complementation experiments of MG1655 Δ*dnaKJ* mutant transformed with a plasmid encoding DnaK and DnaJ confirmed that the carbon source-specific growth defects observed were indeed DnaKJ-dependent (Table S1).

These results demonstrate that the lack of DnaK has a major effect on bacterial growth on numerous relevant carbon sources, either alone (class III and IV) or via the fine tuning of HSP synthesis (class I and II). We found that the Δ*dnaKJ* and/or *rpoH*^(I54N)^ mutations severely affected growth on carbon sources belonging to class I and III but not on carbon sources belonging the class V, although some of these carbon sources share the same entry point in the CM, *i.e.*, D-galactose (class I) and D-glucose (class V) enter at the level of glucose-6-phosphate; D-ribose (class III) and D-xylose (class V) at the level of X5P/R5P; D-mannose (class I), D-sorbitol (class III) and NAG (class V) at the level of fructose-6-phosphate (Fig. S3). This suggests that DnaK could act at the first steps of utilization of these compounds, perhaps at the level of their transport. The previously observed membrane localization of DnaK in *E. coli* is in line with such proposed DnaK function^26^. In the case of mannose, previous data indicate that the absence of DnaK induces the repression of the *manXYZ* operon encoding for the mannose transporter^27^. Yet, DnaK also interacts directly with the ManX subunit of the mannose permease at a late step of its folding process, thus suggesting that assistance by DnaK might also take place at the level of transporter assembly^8^. The fact that glucosamine also uses the mannose transporter to enter the cell suggests a similar mechanism^28^.

### DnaK affects the extracellular accumulation of metabolic by-products

**Figure 3:**
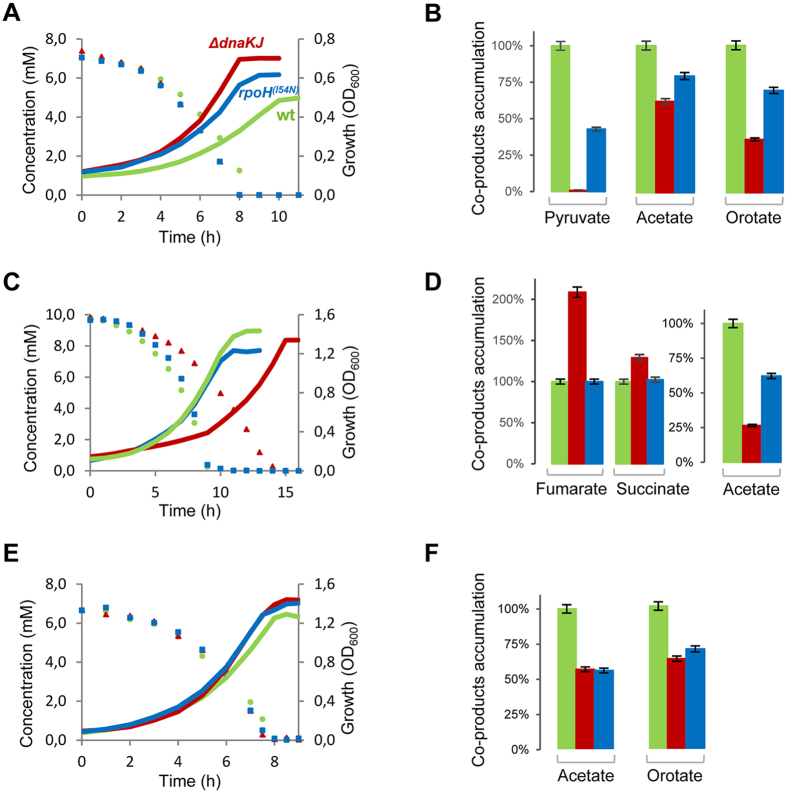
Effect of DnaK on carbon sources utilization and extracellular accumulation of metabolic products. Growth kinetics and carbon utilization monitored for MG1655 wild type (green), Δ*dnaKJ* (red) and *rpoH*^(I54N)^ (blue) grown on lactate (**A**), malate (**C**) and glucose (**E**). Extracellular accumulation of metabolic compounds detected during growth MG1655 wild type (green), Δ*dnaKJ* (red) and *rpoH*^(I54N)^ (blue) on lactate (**B**), malate (**D**) and glucose (**F**) corresponds to molar yields (mol of by-products formed/mol of carbon sources consumed) relative to those measured for the wild type. Growth was monitored by optical density measurement at 600 nm (OD_600_) and compounds in culture supernatant were quantified by 1D ^1^H-NMR every 30 min.

To further characterize the impact of DnaK in the CM, we next analyzed the different metabolites, *i.e.,* the carbon substrates and the metabolic by-products, present in culture supernatants using an untargeted quantitative NMR approach. Analyses were performed for each time point on culture supernatants collected during growth of the three strains on each of the 21 carbon sources. Representative sets of substrate consumption and bacterial growth experiments obtained on lactate, malate and glucose are shown as examples in Fig. 3A,C and E. Substrate consumption, growth and specific consumption rates (q_s_) for each strain on each carbon source are summarized in Table S2. Generally q_s_ vary accordingly to growth rates, leading to similar biomass yields for the three strains. Yet, in the case of pyruvate and lactate, q_s_ are lower for both mutants although growth rates are significantly higher. This suggests that an increased level of HSPs induces an increased biomass yield on these carbon sources. Analysis of metabolic by-products revealed that pyruvate accumulates in the supernatant when wild type was grown on lactate Fig. 3B. In contrast, we found that the Δ*dnaKJ* mutant accumulates significantly less pyruvate when compared to the wild type, suggesting that pyruvate is more efficiently consumed in the absence of DnaK. Interestingly, three of the Δ*tig* Δ*dnaKJ* multicopy suppressors, namely LdhA, Lpd and PykF play a role in pyruvate production and utilization, thus suggesting that DnaK could regulate pyruvate utilization within the cell. Yet a direct involvement of DnaK at the pyruvate node remains to be determined.

More generally, analysis of metabolic by-products revealed that acetate and orotate were both efficiently detected in culture supernatants (Table S2), except for cells grown on glycerol. Acetate is known to be excreted during *E. coli* growth^29^ and accumulation of orotate is generally explained by the reading frame shift in *rphE* that is present in *E. coli* MG1655, which generates a bottleneck within the pyrimidine biosynthesis pathway^30^. Molar yields relative to substrates were also calculated using substrate consumption and metabolic by-product production (Table S2). All together, we found that DnaK significantly impacts the excretion patterns of coproducts. The production of extracellular metabolic compounds (*i.e*., acetate, orotate, pyruvate, succinate and fumarate) for both mutants relative to the wild type grown on lactate, malate and glucose, respectively members of classes II, III and V, are shown in Fig. 3B,D and F. Note that orotate was not detected during growth on malate. Remarkably, the molar production yields of acetate and orotate are always lower for both mutants when compared to the wild type. Although acetate and orotate accumulation is generally considered growth rate dependent^31^, we found that this does not always apply to both *dnaK* and *rpoH* mutants. Indeed, excretion of acetate and orotate is significantly reduced in both mutants when compared to the wild type, while their growth rates are similar or even higher on class V and II carbon sources, respectively. These data reveal a major role of DnaK and the HSR in the metabolism of acetate and orotate.

### DnaK imposes a hierarchal order of carbon utilization when multiple carbon sources are present

**Figure 4:**
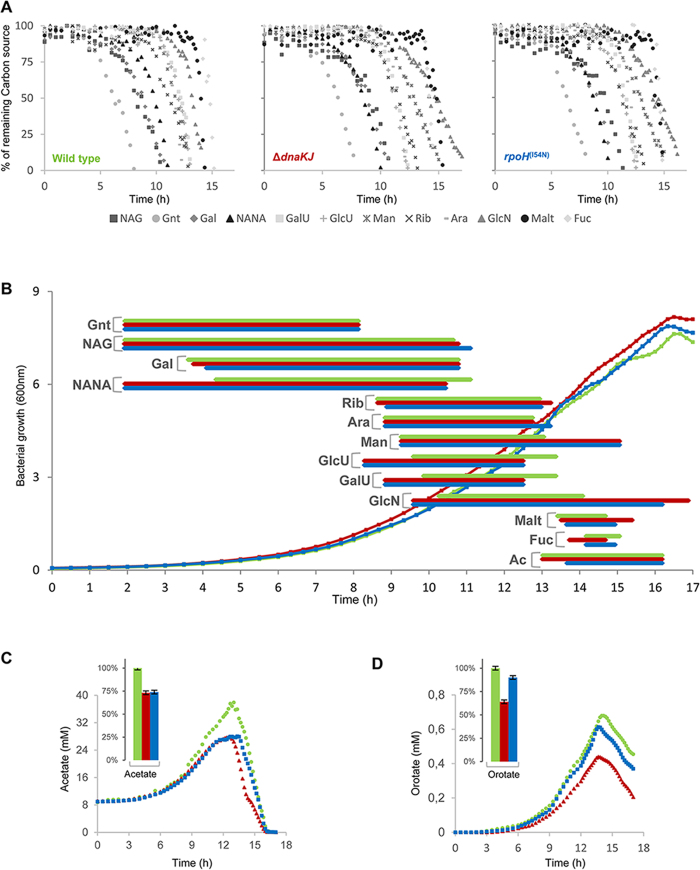
DnaK-dependent adaptive growth in complex mixture of carbon sources known to be present in intestinal environment. (**A**) Carbon sources consumption by MG1655 wild type (left panel), Δ*dnaKJ* (middle panel) and *rpoH*^(I54N)^ (right panel) in a chemically defined medium supplemented with 13 carbon nutrients (0.5 g/l each). Carbon sources in culture supernatants were quantified by ^1^H 1D-NMR. (**B**) The period of time where consumption of the indicated sugar began and was completed, are depicted by horizontal bars, green for the *E. coli* K-12 MG1655 wild type, red for Δ*dnaKJ* mutant and blue for *rpoH*^(I54N)^mutant. Abbreviations: gluconate (Gnt), N-acetyl-glucosamine (NAG), galactose (Gal), N-acetyl-neuraminate (NANA), ribose (Rib), arabinose (Ara), mannose (Man), glucuronate (GlcU), galacturonate (GalU), glucosamine (GlcN), maltose (Malt), fucose (Fuc), and acetate (Ac). acetate (**C**) and orotate (**D**) concentrations in culture supernatant during growth in the complex mixture of carbon sources and % of extracellular accumulation (mol of by-products formed/mol of carbon sources consumed) relative to those measured for the wild type (left insets).

Our results show that Δ*dnaKJ* mutation can have a profound effect on *E. coli* growth according to the carbon source available. Yet, in its main ecological niche *i.e.,* the mammalian intestine, *E. coli* generally has access to several carbon sources available to support bacterial growth and colonization of the intestine. Accordingly, it has been shown that *E. coli* can efficiently co-metabolize several sugars and that its survival and colonization abilities are defined by nutrient availability and consumption^32^. Therefore, we next investigated whether DnaK impacts carbon consumption when several carbon sources are simultaneously present in the same growth medium. To this aim, we followed bacterial growth, carbon consumption and excreted compounds of the three strains at 30 °C in a medium containing 13 different carbon sources, *i.e*., NAG, gluconate, D-galactose, N-acetyl-neuraminate (NANA), D-galacturonate, D-glucuronate, D-mannose, D-ribose, L-arabinose, glucosamine, maltose, L-fucose and acetate (0,5 g/l each), as described by Fabich and colleagues^32^. Of note, only 4 out of 13 carbon sources present in the mix, D-galactose, D-mannose, D-ribose, glucosamine did not support growth of the Δ*dnaKJ* mutant when present as sole carbon sources (Fig. 2). The results from Fig. 4A and B clearly show important differences between the strains. In this case, the order of nutrient preference was determined at 50% of carbon consumption for each carbon source. The preference order for *E. coli* MG1655 wild type, *i.e*., gluconate > NAG = galactose > NANA > ribose > arabinose = mannose = glucuronate = galacturonate > glucosamine > maltose > fucose, is similar to that determined by Fabich *et al*.^32^, except for gluconate and NAG for which the preference order is reversed. The temperature of growth used in this work (30 °C versus 37 °C in ref. 32) could account for such difference. Although no significant difference in the order of carbon sources consumption could be observed between Δ*dnaKJ* and *rpoH*^(I54N)^, the preference order of the two mutants (gluconate > NANA = galactose > NAG > glucuronate = galacturonate = ribose = arabinose > mannose > maltose = fucose > glucosamine) is significantly different than the one observed for the wild type, with NANA, glucuronate and galacturonate being consumed earlier by both mutants (Fig. 4A and B). Together these results indicate that in the presence of multiple carbon sources DnaK significantly contributes to the establishment of a hierarchical order of carbon utilization, mainly via its regulatory effect on the synthesis of HSPs.

**Figure 5:**
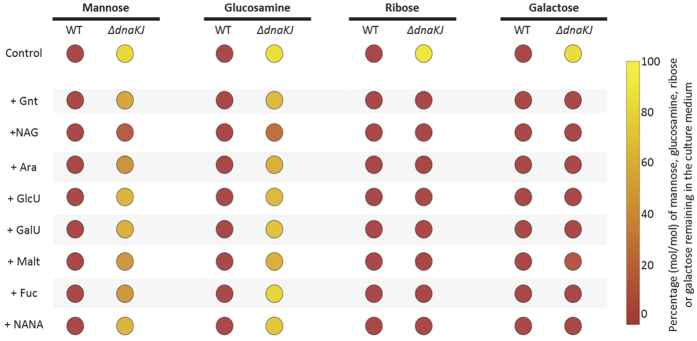
Mannose, glucosamine, ribose and galactose utilization in presence of one additional carbon source. Cultures of MG1655 wild type and Δ*dnaKJ* grown in LB media overnight at 30 °C were washed and transferred in minimal M9 based-medium supplemented with glucose (2.7 g/L). Before cells enter the stationary phase, cells were washed and then inoculated at an initial OD_600_ of 0.1 in M9 based-medium supplemented with binary mixture of two carbon sources one of which was mannose, glucosamine, ribose or galactose. Concentration of each carbon source in the medium was 1 g/L. Cultivations were performed in triplicate using a bioreactor block in an automatic high throughput fluxomic workstation (Freedom EVO 200, TECAN, Switzerland). Samples of culture supernatants were collected when cells stopped growing and analyzed by 1D 1H NMR, to determine the proportion of mannose, glucosamine, ribose or galactose remaining in the medium. All analyses showed exhaustion of all the others carbon sources. Abbreviations: gluconate (Gnt), N-acetyl-glucosamine (NAG), N-acetyl-neuraminate (NANA), arabinose (Ara), glucuronate (GlcU), galacturonate (GalU), maltose (Malt), fucose (Fuc).

This work also reveals that the Δ*dnaKJ* mutant can fully consume ribose and galactose within the mixture, while it could not when they were present as sole carbon sources. The same observation was made for the *rpoH*^(I54N)^ mutant in the presence of galactose. Similarly, the two mutants are now capable of consuming mannose and glucosamine, both carbon sources that normally do not support growth of the mutants as sole carbon sources, albeit less efficiently than the wild type strain (Fig. 4A and B). The fact that the simultaneous presence of multiple carbon sources allows the utilization of DnaK-dependent substrates (Fig. 2) suggests that certain metabolic pathways and/or transporters in place somehow buffer for the absence of DnaK^28^,^33^. To address such a hypothesis, we monitored mannose, glucosamine, ribose or galactose utilization by both wild type and Δ*dnaKJ* cells grown in binary mixtures of carbon sources containing mannose, glucosamine, ribose or galactose, together with one of each of the permissive carbon sources present in the multiple carbon source experiment from Fig. 4. We found that in the absence of DnaK, the presence of NAG, but none of the other carbon sources, could specifically trigger the utilization of both mannose and glucosamine (Fig. 5). Since the mannose uptake system, known to support the transport of both mannose and glucosamine^34^, is depleted in the absence of DnaK, it is likely that the presence of NAG might specifically induce the production of non-specific transporter(s) for mannose and glucosamine. In sharp contrast, the utilization of both ribose and galactose by the Δ*dnaKJ* mutant was triggered by each of the other carbon sources tested here (Fig. 5). Although the precise cellular mechanism involved remains unknown, it shows that at least one other carbon or energy source is required to initiate utilization of these two compounds in the absence of DnaK. Together these results strongly suggest that different metabolic processes can support the utilization of DnaK-dependent carbon sources.

It is believed that acetate is utilized when consumption of preferential, high energy carbon sources is completed^29^. In the case of the wild type strain, acetate utilization is concomitant with a full consumption of gluconate, galactose, NANA, NAG, arabinose, ribose, mannose, galacturonnate, glucoronate and glucosamine, while fucose and maltose are not yet consumed (Fig. 4B). For both mutants, acetate consumption initiates even before mannose and glucosamine consumption was completed, thus indicating that acetate is not the last carbon source to be consumed (Fig. 4B and C). Finally, the mutants also accumulate lower amount of orotate and acetate, as observed on single carbon sources (Fig. 3), thus further supporting a major role of DnaK in acetate and orotate production and/or excretion, independently of carbon availability and utilization.

### DnaK severely impacts acetate metabolism

The phenotypic analysis of Δ*dnaKJ* and *rpoH*^(I54N)^ mutants (Figs 2 and 3) and the multicopy suppression of both Δ*tig* Δ*dnaKJ* and Δ*dnaKJ* mutants by AckA (Fig. 1) reveal major links between DnaK and acetate metabolism. To further investigate such interplay, we monitored bacterial growth either at high acetate concentration (40 mM), in which the phosphate acetyltransferase Pta and AckA pathway forms the major route for acetate utilization, or at low acetate concentration (6 mM), where acetate is mostly scavenged by the acetyl-coA synthetase Acs^35^. At high acetate concentration, the growth rate of the Δ*dnaKJ* strain is severely reduced when compared to the wild type and the *rpoH*^(I54N)^ strains, and is directly correlated with a lower acetate consumption rate (Fig. S4). This is in agreement with both the multicopy suppression by AckA and the reduced cellular concentration of AckA found in the absence of DnaK (Fig. 1)^8^. In contrast, at low acetate concentration we observed a faster growth for both mutants when compared to the wild type, which was correlated with a higher acetate consumption rate (Fig. S4). This indicates that the absence of DnaK specifically slows down acetate utilization via the AckA-Pta pathway and, through the induction of HSPs, likely facilitates acetate utilization via Acs. Of note, overexpression of either Pta or Acs did not suppress the growth defect of the double Δ*tig* Δ*dnaKJ* mutant (Fig. S4), suggesting that AckA could be the only limiting enzyme for acetate production/utilization in such a chaperone deficient context. Interestingly, AckA, which is responsible for the reversible conversion of AcP into acetate, regulates the intracellular concentration of AcP, known to have global regulatory functions in *E. coli*.^29^ Since AcP, was shown to stimulate protein disaggregation and degradation under stress conditions^36^,^37^, these data suggest that DnaK could additionally maintain protein homeostasis by modulating the levels of cellular AcP.

### DnaK modulates carbon metabolic flux distribution within cells

Our data suggest that the proper functioning of the CM might be affected by the absence of DnaK. However, despite a significant decrease in acetate excretion, the Δ*dnaKJ* mutation does not affect growth on carbon sources belonging to the class V, although their utilization massively involves the CM. One possible explanation in these cases, is that the Δ*dnaKJ* mutant undergoes a functional remodeling of the CM, which eventually leads to a silent growth phenotype^38^. To investigate such possible phenomenon, metabolic flux maps of the wild type and the two mutants were established using a steady-state ^13^C-labeling experiment with a mixture of 80% 1-^13^C and 20% U-^13^C-glucose as sole carbon source at the permissive temperature of 30 °C. Of note, Calloni and coworkers^8^ also used glucose to determine the DnaK interactome and most current flux analyses have been performed on glucose as well, thus providing comparable data sets. In this case, labeled proteinogenic amino acids and extracellular metabolites were analyzed by 1D- and 2D-NMR methods (Table S3), and flux distribution within the central metabolism pathways was calculated from each isotopic dataset^39^.

**Figure 6:**
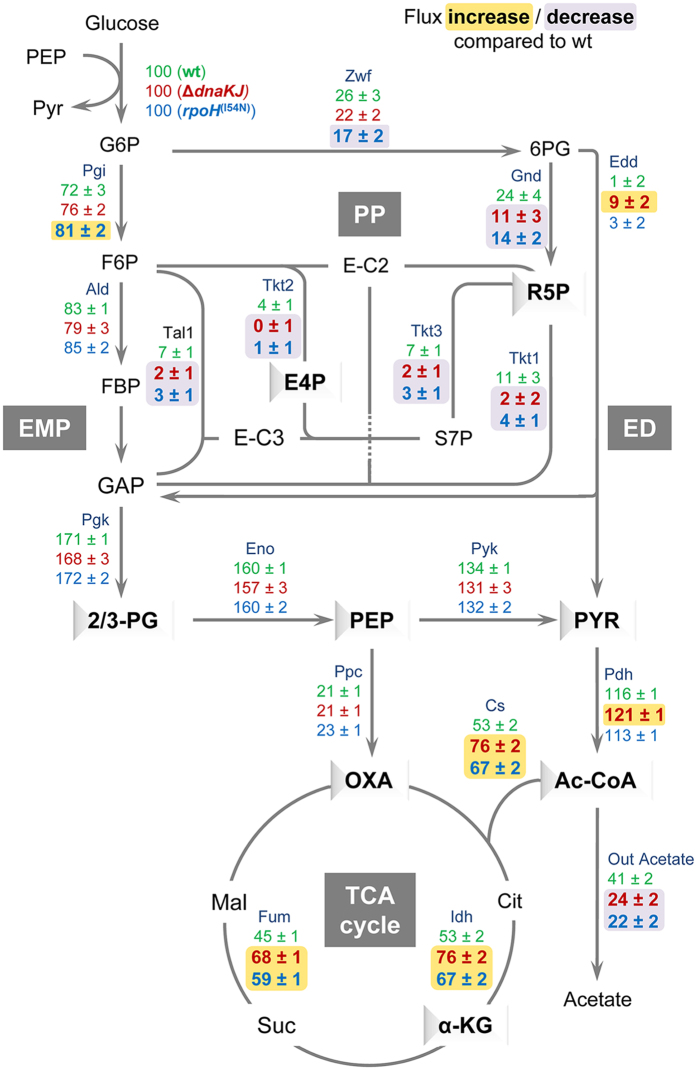
DnaK modulates metabolic flux distribution. Flux distributions within central metabolism for the three strains: upper values in green, wild type; middle values in red, Δ*dnaKJ* mutant; lower values in blue, *rpoH*^(I54N)^mutant. Underlined values indicate a flux increase (yellow) or decrease (purple) compared to the wild type, respectively. The three different strains were grown at 30 °C in minimal medium supplemented with a mixture of 80% [1-^13^C] and 20% [U-^13^C] glucose. All fluxes are presented as a molar percentage of the specific glucose uptake rate. The data are presented as flux ± 95% Confidence Interval (CI), the latter being determined by Monte-Carlo-based sensitivity analysis. Metabolite precursors for amino acid biosynthesis are depicted in relief. Abbreviations: fructose-1,6-bisphosphate (FBP), citrate (Cit), combined pool of 2- and 3-phosphoglycerate (2/3-PG), erythrose-4-phosphate (E4P), sedoheptulose-7-phosphate (S7P), Glucose-6-phosphate 1-dehydrogenase (zwf), Glucose-6-phosphate isomerase (Pgi), 6-phosphogluconate dehydrogenase (Gnd), Phosphogluconate dehydratase (Edd), fructose-bisphosphate aldolase (Ald), Transaldolase (Tal), Transketolase (Tkt), Phosphoglycerate kinase (Pgk), Enolase (Eno), Pyruvate kinase (Pyk), Phosphoenolpyruvate carboxylase (Ppc), Citrate synthase (Cs), Pyruvate dehydrogenase (Pdh), Fumarase (Fum), Isocitrate dehydrogenase (Idh). All the other abbreviations are from Fig. 1

The flux map obtained for the wild type strain (Fig. 6 and Table S4) showed that glucose is mainly catabolized via the EMP pathway. The fraction of glucose catabolized via the PP pathway represents 24% and only 1% via the ED pathway. At the end, 53% of the glucose enters the TCA cycle via the citrate synthase (Cs); the anaplerotic flux (Ppc) represents 21%. The flux distribution was similar to that previously described for *E. coli* wild type MG1655 at 37 °C, indicating that lowering the temperature does not detectably modify the functioning of the CM, although the growth rate is significantly lower (0.37 h^−1^ at 30 °C versus 0.56 h^−1^ at 37 °C).

Remarkably, metabolic fluxes through the PP pathway are significantly reduced for both mutants when compared to the wild type (Fig. 6 and Table S4). Of note, TalB, which is a robust suppressor of both Δ*tig* Δ*dnaKJ* and Δ*dnaKJ* mutants, is involved in the PP pathway. Whether TalB is a limiting factor in this case is not known. In the case of *rpoH*^(I54N)^, the relative flux through the EMP pathway is higher than the ones observed for the wild type and the Δ*dnaKJ* mutant, thus reflecting the reduced flux in the PP pathway for this mutant. The split ratio between the PP and the ED pathways for the Δ*dnaKJ* mutant is significantly higher when compared to the two other strains, with a flux through ED of 9%, thus leading to a corresponding decrease of the relative flux in the PP pathway (from 24% for the wild type to 11% for the mutant). Therefore, in the presence of glucose, the contribution of the PP pathway is significantly lower in the two mutants compared to the wild type strain due to a higher contribution of the EMP pathway for the *rpoH*^(I54N)^ mutant, and of the ED pathway in the case of the Δ*dnaKJ* mutant. Intriguingly, a comparable decrease of fluxes in the PP pathway was found for the *lpd* mutant in *E. coli*, further supporting the link between DnaK and the suppressor Lpd described above^40^. Importantly, a significant flux increase in the TCA cycle was observed for both Δ*dnaKJ* and *rpoH*^(I54N)^ mutants. In this case, fluxes that enter the TCA cycle were about 30% and 40% higher for the *rpoH*^(I54N)^ and Δ*dnaKJ* mutants, respectively. Correspondingly, the flux increase in the TCA cycle correlates with a major decrease in acetate production (about 50%).

In sum, these flux data show that in the absence of DnaK there is significant carbon flux redistribution in the PP and ED pathways, in the TCA cycle and in the acetate production. A similar rearrangement is observed in the *rpoH*^(I54N)^ mutant, except for the ED pathway, suggesting that DnaK’s impact on fluxes remodeling predominantly occurs via the control of HSP synthesis.

Carbon fluxes distribution was also used to estimate the energetic fluxes in terms of NADPH and ATP production fluxes (Table S5). For both mutants, we found that there is a default in NADPH production at the level of the PP pathway that is efficiently compensated by the increased fluxes in the TCA cycle at the level of isocitrate dehydrogenase (Idh), which is involved in NADPH synthesis. This is in complete agreement with the fact that biomass yields and growth rates are comparable for the three strains (Fig. 2 and Table S2). In addition, increased fluxes in the TCA cycle also resulted in higher ATP production fluxes, which appeared to be more pronounced in the case of the Δ*dnaKJ* mutant (Table S5; see below).

## Discussion

In this work, we have combined genetic, metabolomic and fluxomic approaches to investigate the role of the stress-responsive chaperone DnaK/HSP70 in the CM. We have identified several enzymes of the CM that partially compensate for the lack of DnaK and relevant carbon sources whose utilization was differentially affected by DnaK. In addition, we found that DnaK function significantly contributes to the establishment of a hierarchical order in the utilization of carbon sources when multiple carbon sources are simultaneously available during growth, thus indicating that DnaK modulates the uptake and consumption of available substrates. Moreover, we demonstrated that DnaK differentially affects the excretion of main metabolism coproducts, including acetate, and significantly impacts metabolic flux distribution, mainly by modulating fluxes through the TCA cycle. Together these results demonstrate that DnaK multitasking chaperone function is critical for the proper functioning of the CM, acting at several key steps of carbon utilization.

**Figure 7:**
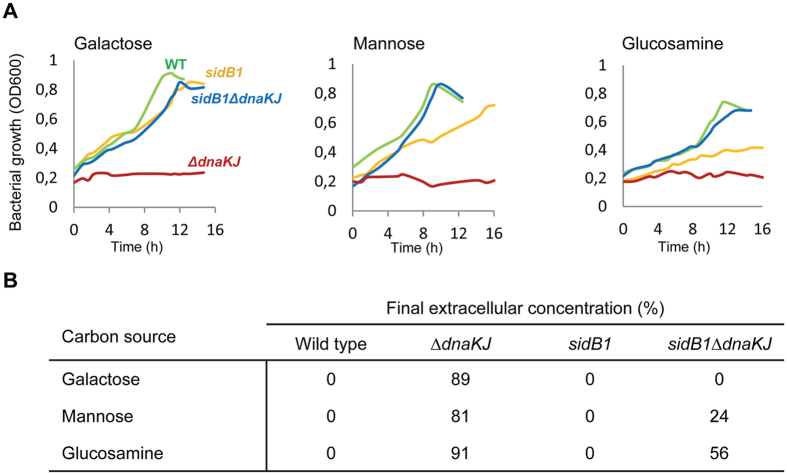
Destabilizing σ^32^ in the absence of DnaK partially restores growth on class I carbon sources. (**A**) Representative growth curves of MG1655 wild type (green), Δ*dnaKJ* (red), *sidB1* (blue) and *sidB1* Δ*dnaKJ* (yellow) on class I carbon sources, namely galactose, mannose, glucosamine. Strains were first grown at 30 °C in minimal M9 medium supplemented with glucose (2.7 g/L). Mid-log phase cultures were washed and then inoculated at an initial OD = 0.2 in M9 medium supplemented with 1 g/L galactose, mannose, glucosamine. Cultivations were performed in triplicate using a bioreactor block in an automatic high throughput fluxomic workstation (Freedom EVO 200, TECAN, Switzerland). (**B**) Samples of culture supernatants were collected when cells stopped growing and analyzed by 1D ^1^H NMR, to determine the concentration of each carbon source remaining in the medium.

In many cases, we found that the effect of DnaK on the CM was directly connected to its role in downregulating the HSR. Remarkably, induction of HSPs either facilitates (class II carbon sources) or limits (class I carbon sources) the utilization of certain carbon sources (Fig. 2), and significantly modifies the order of carbon source consumption (Fig. 4). This indicates that a fine-tuning of HSP synthesis by endogenous DnaK level might represent a highly efficient way to successfully adapt to carbon source changes or limitation. Interestingly, we found that the *sidB1* mutation known to partially destabilize σ^32^ independently of its interaction with DnaK and thus to reduce the endogenous level of HSPs in a *dnaK* mutant^41^ significantly restored growth and utilization of class I carbon sources that are normally not efficiently used by both Δ*dnaKJ* and *rpoH*^(I54N)^ mutants (Fig. 7). This strongly suggests that the loss of utilization of these carbon sources is indeed mediated by DnaK’s regulation of cellular HSP level.

The previously observed catabolic repression of the *dnaKJ* operon further supports such an adaptive model involving DnaK^18^. The fact that NANA, which is one of the most abundant components of mucins^42^, is consumed earlier by both Δ*dnaKJ* and *rpoH*^(I54N)^ mutants (Fig. 4) additionally suggests that induction of HSPs might confer a substantial advantage during bacterial colonization^43^. Noticeably, our data also revealed that induction of HSPs helps bacteria to grow on low acetate concentration (Fig. S4), thus suggesting that when preferential carbon sources are consumed, a DnaK-dependent induction of HSPs might also facilitate the utilization of metabolic co-products, such as acetate and pyruvate, to promote bacterial growth^29^.

Although this work clearly shows that DnaK, either alone or via the control of HSP synthesis, can act as a key modulator of the CM, the fact that the cellular abundance of a significant number of proteins is altered in a Δ*dnaKJ* mutant^8^, suggests that some of the observed changes in the CM could be indirect consequences of the absence of DnaK or of the resulting constitutive activation of the heat-shock response, both known to induce large-scale transcriptional responses. This is in agreement with the proposed role of the stress-responsive DnaK chaperone as a major hub in the cellular proteostasis network^8^.

Previous studies showed that under stress conditions affecting cellular protein homeostasis, DnaK chaperone tasking is efficiently rerouted towards accumulating protein aggregates, subsequently leading to the rapid stabilization of σ^32^ and a prolonged induction of HSPs, which includes major chaperones and proteases known to cooperate with DnaK^11^,^44^. Our data further extend this model, suggesting that the capture of DnaK by aggregates could additionally provoke significant rearrangements of the metabolism, which might be crucial for cell survival under such circumstances. This includes changes in the CM regulatory networks, in the uptake of specific compounds, in the production of certain metabolites, and in the redistribution of carbon fluxes within the cell. Remarkably, the unexpected increase in metabolic fluxes through the TCA cycle induced by both the lack of DnaK and the induction of HSPs, suggests that titration of DnaK and the subsequent induction of HSPs might provoke a rapid increase in the flux of ATP production. Such phenomenon could well reflect the transient increase in cellular ATP that was previously observed following high temperature stress^45^. Since a significant fraction of major chaperones and proteases involved in the cellular defense against protein aggregation are indeed ATP-dependent^1^, it is reasonable to assume that such a DnaK-dependent process might represent a highly efficient way to accumulate rapid surplus of energy that becomes crucial for bacterial survival under proteostasis collapse^8^. Further work is warranted to elucidate such a possible DnaK-dependent response that couples protein homeostasis to ATP production in bacteria.

## Methods

### Bacterial strains, phages, culture conditions and plasmid constructs

The MC4100 mutant derivatives Δ*dnaKJ*::Kan^R^, Δ*tig*::Cm^R^ Δ*dnaKJ*::Kan^R^ 14, *rpoH*^(I54N)25^ have been previously described. Mutations described in this study were moved into the MG1655 genetic background by bacteriophage P1-mediated transduction. The W3110 Δ*dnaK*52::Cm^R^ *thr*::Tn*10* has been previously described^19^. The Δ*ackA*::Kan^R^, Δ*ldhA*::Kan^R^, Δ*lpd*::Kan^R^, Δ*pykF*::Kan^R^ and Δ*talB*::Kan^R^ mutant alleles were obtained from strain JWK2293, JWK1375, JWK0112, JWK1666 and JWK0007, respectively (Keio collection). The Δ*csrB*Δ*csrC::*Kan^R^ is a kind gift of Catherine Turlan and Agamemnon Carpousis (Toulouse). The MG1655 *sidB1* derivatives were constructed as follows. The *zhf-50*::Tn*10* (TetR) allele from CAG18450^46^ was first moved into MC4100 *sidB1* strain using P1 transduction and selection at 30 °C on LB agar plates supplemented tetracycline (from Bernd Bukau via Costa Georgopoulos^41^). Following sequencing *of the sidB* allele, a new P1 lysate was prepared and used to transduce the *sidB1* allele linked to *zhf-50*::Tn*10* (TetR) into MG1655 following selection at 30 °C on LB agar plates supplemented tetracycline. Finally, the Δ*dnaKJ*::KanR mutant allele was then moved into MG1655 *sidB*^*+*^*zhf-50*::Tn*10* (TetR) and MG1655 *sidB1 zhf-50*::Tn*10* (TetR), thus resulting in a set of isogenic MG1655 derivatives. Bacteria were grown in LB or M9 based medium with specific carbon sources (see below) supplemented when necessary with chloramphenicol (10 μg/ml), kanamycin (50 μg/ml), ampicillin (50 μg/ml) and tetracycline (15 μg/ml).

Plasmid pSE380ΔNcoI is a derivative of pSE380 from Invitrogen (www.addgene.org/vector-database/4063/) in which a small region of the multi-cloning site (nt 268 to 306 that includes the NcoI to BstB1 restriction sites) has been deleted, leaving an EcoRI site directly downstream the original ribosome-binding site. This leads to the following sequence: CACAGGA**GAATTC**AGCA (ribosome-binding site underlined and EcoRI site bold)^14^. To construct the high-copy number plasmids pSE-AckA, pSE-LdhA, pSE-Lpd, pSE-PykF, pSE-TalB, pSE-csrB, pSE-csrC, pSE-Pta, pSE-Acs the 1203-bp *ackA*, 990-bp *ldhA*, 1425-bp *lpd*, 1413-bp *pykF*, 954-bp *talB*,369-bp *csrB*, 245-bp *csrC*, 2145-bp *pta* and 1959-bp *acs* coding sequences were PCR-amplified using appropriate primers listed in Table 1 and MG1655 genomic DNA as template. The PCR fragments were digested with EcoRI and XhoI and cloned into pSE380ΔNcoI previously digested with the same enzymes.

### Genetic selection

Genetic suppressors of the Δ*tig* Δ*dnaKJ* double mutant were isolated as follows. A pMPMA2–based *E. coli* multicopy genomic library (laboratory collection) was transformed into MG1655 Δ*tig* Δ*dnaKJ* and selection was performed at 35 °C on LB agar plates supplemented in ampicillin. From approximately 70 000 transformants tested, ninety two plasmids capable of suppressing the growth defect were first extracted and retransformed into the same strain in order to confirm suppression. The thirty two suppressors that passed the second selection were then sequenced. At the end we have identified 19 suppressor fragments encoding proteins that belong to diverse cell pathways (metabolism, protein processing, translation and transcription) or have unknown functions. Interestingly, six of the suppressor fragments contain genes involved in the central metabolism of the cell (Fig. 1).

### Bacterial growth on specific carbon sources

Overnight cultures in LB at 30 °C were diluted 1/50, grown in M9 based medium with glucose (2 g/l) until mid-log phase (OD_600_ 0,8) at 30 °C, washed with M9 salts and inoculated at OD_600_ 0,1 and grown aerobically in baffled Erlenmeyer flask containing M9 based medium containing independently L-arabinose (1.2 g/l), D-glucose (1.2 g/l), malate (1.2 g/l), succinate (1.2 g/l), maltose (1.2 g/l), N-acetyl-glucosamine (1.2 g/l), glycerol (1.2 g/l), L-fucose (0.7 g/l), pyruvate (0.7 g/l), D-xylose (0.7 g/l), D-galactose (0.7 g/l), D-glucosamine (0.7 g/l), D-mannose (0.7 g/l), D-ribose (0.7 g/l), D-sorbitol (0.7 g/l), L-rhamnose (0.7 g/l), N-acetyl-neuraminate (0.7 g/l), L-lactate (0.7 g/l), D-glucuronate (0.7 g/l), galacturonate (0.7 g/l), fumarate (0.7 g/l) or acetate (0.35 g/l or 2.5 g/l). Carbon source references are listed in Table 2. Samples were taken every 30 min for 16 hours during bacterial growth on the different carbon sources described above. For each time point, OD_600_ was monitored, 500 μl of culture was filtered (Sartolon polyamide 0.2 μm, Sartorius) and stored at −20 °C before NMR analysis. In the case of the multiple carbon source assay the second inoculation was performed to an OD_600_ of 0,05 and grown in M9 containing N-Acetyl glucosamine, gluconate, galactose N-acetyl-neuraminate, galacturonate, glucuronate, mannose, ribose, arabinose, glucosamine, maltose, fucose and acetate (0,5 g/l each). Samples were taken every 30 min for the first 6 hours then every 20 min until 15 hours and every 10 min for the last two hours. Each time OD_600_ was monitored and sample for NMR analysis were taken.

### Sampling and NMR analysis of extracellular metabolites

The main metabolic products which accumulated during growth in the extracellular medium were measured by NMR spectroscopy. Samples were analyzed by quantitative ^1^H 1D-NMR at 298 K, using 30° pulse and a relaxation delay of 10 s with an advanced 800 MHz spectrometer (Bruker, Rheinstetten, Germany). A total of 64 scans were accumulated after 8 dummy scans. Molar yields have been calculated at the time of exhaustion of the primary carbon source. Spectra collected from samples of the 13 carbon source experiments contained at least one isolated peak for each carbon source from which the quantification of the compound could be performed unambiguously. This analysis was performed at 280 K.

### Calculation of fluxes of ATP and NADPH production

NADH, NADPH and FADH_2_: total production of each reduced cofactor was calculated by adding fluxes of all reactions which produced the reduced cofactor. ATP: production of ATP via substrate-level phosphorylation was calculated by adding all the fluxes measured for all ATP producing reactions in carbon metabolism, and subtracting the fluxes of all ATP-consuming reactions. The ATP produced from NADH and FADH_2_ via oxidative phosphorylation was estimated assuming a P:O ratio of 1.75 for both redox cofactors^47^.

### ^13^C-metabolomic Flux analysis: sampling, analysis of proteinogenic amino acids by two-dimensional NMR and flux calculation

Overnight cultures of MG1655 wild type, Δ*dnaKJ* and *rpoH*^(I54N)^ strains grown in LB at 30 °C were diluted 1/50 and grown in baffled Erlenmeyer flask containing M9 based medium with a mixture of 80% [1-^13^C] glucose and 20% [U-^13^C] glucose (Table S2) until mid-log phase (OD_600_ 0,8) at 30 °C. Cells were washed once with M9 salts inoculated to an OD_600_ of 0,05 and incubated at 30 °C a second time in baffled Erlenmeyer flask containing M9 based medium with the same glucose labeled mixture at 30 °C to minimize the effect of unlabeled carbon. Note that the ratio of (1-^13^C) and (U-^13^C) glucose used in this experiment was determined and optimized using the software Isodesign^48^.

Culture samples were collected for NMR analyses at an OD_600_ of 1 to ensure both isotopic and metabolic steady-state conditions. In this case, 9 ml of solvent H_2_O/Methanol/Acetonitrile (20:40:40) was added to 1 ml of cell culture to quench the metabolism and samples were stored at −20 °C for 15 min. The mixture was then centrifuged at 12,000 *g* at −20 °C for 5 min. Then, the 400 mg of harvested cells were incubated in 5 ml of 6 M HCl at 105 °C for 24 h for metabolite extraction. The acid was removed by evaporation (SC110A SpeedVac Plus), and labile protons were exchanged three times with deuterium by successive resuspension in 2 ml of D_2_O 99.8% (EurisoTop), and the hydrolysate was finally resuspended in 600 ml of D_2_O before analysis.

Analyses of samples and flux calculation were performed as described by Millard and coworkers (Millard *et al*., 48). Briefly, NMR spectra of samples of proteinogenic amino acids were recorded with an Advance 500-MHz spectrometer (Bruker) at 286 K. The specific enrichments were quantified using a ZQF–TOCSY (zero quantum filter–total correlation spectroscopy) sequence as described by Massou and coworkers^49^. The positional isotopomers were quantified using an HSQC (heteronuclear single quantum correlation) sequence as also described by Massou and coworkers^50^. Spectra were processed using TopSpin 2 (Bruker)^49^,^50^ and isotopomers of accumulated compounds in the extracellular medium, such as acetate and formate, were quantified by one-dimensional ^1^H NMR using the 800 MHz spectrometer. Flux calculations were performed with influx_s (Sokol *et al*., 2012). The metabolic network implemented in the FTBL model included all major reactions of the central carbon metabolism: glucose uptake, EMP, ED and PP pathways, TCA cycle, acetate production and amino acid biosynthesis pathways. Precursor requirements for biomass formation were determined according to the molecular composition of *E. coli*.^51^ and the measured growth rate. Metabolic fluxes were estimated by minimizing the variance-weighted sum of square residuals between the experimental and simulated isotopic data using the NLSIC algorithm implemented in influx_s, correlation between experimental and simulated isotopic data are shown in Fig. S5. To investigate the sensitivity of metabolic fluxes, standard deviations were estimated using a Monte Carlo procedure. 100 iterations were performed for each dataset. All this procedure has been performed on two independent biological replicates.

### Western Blot Analysis

Western blot were performed has described earlier^19^. A rabbit antibody against Lon, a mouse antibody against DnaK, a rabbit antibody against GroEL, a rabbit antibody against DnaJ and a rabbit antibody against sigma 32 were used as primary antibodies, and HRP-conjugated rabbit IgG (1:5,000; Sigma) or mouse IgG (1:2,000; Sigma) were used as secondary antibodies. Membranes were developed using an ECL Plus (GE Healthcare) with a luminescence analyzer (LAS4000, Fuji).

## Additional Information

**How to cite this article:** Angles, F. *et al*. Multilevel interaction of the DnaK/DnaJ(HSP70/HSP40) stress-responsive chaperone machine with the central metabolism. *Sci. Rep.* **7**, 41341; doi: 10.1038/srep41341 (2017).

**Publisher's note:** Springer Nature remains neutral with regard to jurisdictional claims in published maps and institutional affiliations.

## Supplementary Material

Supplementary Information

Supplementary Dataset

## Figures and Tables

**Table 1:** List of primers for pSE-based cloning.

Primer	Sequence
AckA-for	5′-GCAGAATTCATGTCGAGTAAGTTAGTACTG-3′
AckA-rev	5′-GCACTCGAGTCAGGCAGTCAGGCGGCTCG-3
LdhA-for	5′-GCAGAATTCATGAAACTCGCCGTTTATAG-3′
LdhA-rev	5′-GCACTCGAGTTAAACCAGTTCGTTCGG-3′
Lpd-for	5′-GCAGAATTCATGAGTACTGAAATCAAAAC-3′
Lpd-rev	5′-CTCGAGTTACTTCTTCTTCGCTTTC-3′
PykF-for	5′-GCAGAATTCATGAAAAAGACCAAAATTG-3′
PykF-rev	5′-GCACTCGAGTTACAGGACGTGA ACAG-3′
TalB-for	5′-GCAGAATTCATGACGGACAAATTGACCTC-3′
TalB-rev	5′-GCACTCGAGTTACAGCAGATCGC CGATC-3′
CsrB-for	5′-GCAGAATTCAGGAAATAAGCGAATACTTAAA-3′
CsrB-rev	5′-GCACTCGAGAAGAAAAACTGCCGCGAAG-3′
CsrC-for	5′-GCAGAATTCTTGATTGTTTGTTTAAAGCAAAGG-3′
CsrC-rev	5′-GCACTCGAGCTAACAGAAAGCAAGCAAAG-3′
Pta-for	5′-GCAGAATTCGTGTCCCGTATTATTATGCTG -3′
Pta-rev	5′-GCACTCGAGTTACTGCTGCTGTGCAGAC-3′
Acs-for	5′-GCAGAATTCATGAGCCAAATTCACAAACACAC-3′
Acs-rev	5′-GCACTCGAGTTACGATGGCATCGCGAT-3′

**Table 2:** Carbon sources used in this work.

Carbon Source	CAS	Supplier	Reference
D Mannose	3458-28-4	FLUKA	63579
D-Glucosamine	66-84-2	SIGMA	G4875
D-Galactose	59-23-4	SIGMA	C0750
Sodium pyruvate	113-24-6	SIGMA	P5280
Sodium L-lactate	867-56-1	SigmaAldrich	71718
L-Fucose	2438-80-4	SIGMA	F2252
D-Ribose	50-69-1	SIGMA	R9629
L-Rhamnose monohydrate	10030-85-0	FLUKA	83650
Glycerol	200-289-5	VWR	24387-292
D-Sorbitol	50-70-4	SIGMA	240850
Malic Acid	6915-15-7	SIGMA	240176
Succinic acid	110-15-6	FLUKA	14078
Fumaric Acid	110-17-8	FLUKA	47900
D-Xylose	58-86-6	SAFC	W36,060-0
L-Arabinose	5328-37-0	SIGMA	A3256
Maltose monohydrate	6363-53-7	DUCHEFA	M0811,1000
D-Glucuronic acid sodium salt monohydrate	207300-70-7	SIGMA	G8645
D-Galacturonic Acid	14984-39-5	SIGMA	73960
D-Glucose	50-99-7	SIGMA	G7528
N-Acetyl Neuraminic Acid	131-48-6	SigmaAldrich	A0812
N-Acetyl-D-glucosamine	7512-17-6	SIGMA	A8625
Sodium acetate trihydrate	6131-90-4	VWR	27652,298
D-Glucose 1-^13^C	40762-22-9	EURISOTOP	A03-11813
D-Glucose U-^13^C	110187-42-3	EURISOTOP	13H-178
