# Supplementary material for: Multilevel interaction of the DnaK/DnaJ(HSP70/HSP40) stress-responsive chaperone machine with the central metabolism
Source: Sci Rep. 2017 Jan 27;7:41341. doi: 10.1038/srep41341 (PMC5269706; doi:10.1038/srep41341)
Supplement: Supplementary Information [file srep41341-s1.pdf]

SUPPLEMENTARY INFORMATION

Multilevel interaction of the DnaK/DnaJ(HSP70/HSP40) stress-responsive chaperone machine with the central metabolism

Fréderic ANGLES, Marie-Pierre CASTANIE-CORNET, Nawel SLAMA, Mickaël DINCLAUX, Anne-Marie CIRINESI, Jean-Charles PORTAIS, Fabien LETISSE and Pierre GENEVAUX

A

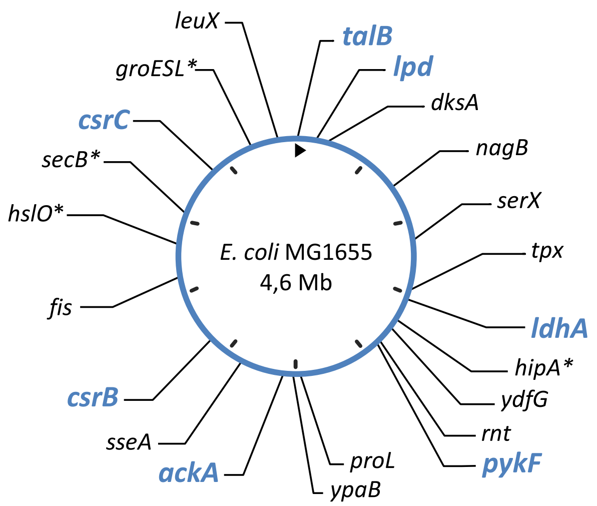

B

Library-based suppressors[a]	Nucleotide position MG1655 genome	Number of clones	Single suppressor gene[b]	Gene ID (EcoCyc)	Suppressor gene function
csrC	4050387-4051659	1	***csrC***	G0-8874	Small RNA inhibits the protein regulator of carbohydrate metabolism CsrA
	4050386-4051748	1			
csrC,yihl	4050386-4052293	2			
	4050386-4052083	1			
ybdJ,hslJ,ldhA	1440920-1444452	1	***ldhA***	G592	D-lactate dehydrogenase; σ^{32} regulon
lpd	126814-130239	1	***lpd***	EG10543	Lipoamide dehydrogenase; catalyzes the transfer of electrons to NAD
yfbU,yfbV,ackA	2411941-2415361	1	***ackA***	EG10027	Acetate kinase
yfbV,ackA	2412507-2415428	1			
yaaJ,talB	6354-9345	2	***talB***	EG11556	Transaldolase B
ydhZ,pykF	1754680-1757316	1	***pykF***	EG10804	Pyruvate kinase I
secB,grxC	3783180-3785184	1	*secB*	EG10937	Molecular chaperone
tpx	1387440-1389221	1	*tpx*	G6660	Thioredoxin 1-dependent thiol peroxidase
gluQ,dksA	158510-160637	1	*dksA*	EG10230	RNA polymerase-binding transcription factor
dusB,fis,yhdJ, yhdU,envR	3410177-3413916	1	*fis*	EG10317	DNA-binding and bending protein involved in nucleoid structure
rnt	1728179-1730740	1	*rnt*	EG11547	Subunit of Rnase T; 3'-5' exonuclease responsible for 3' trimming of stable RNAs
leuX	4495544-4497378	3	*leuX*	EG30053	Leucine tRNA
	4495509-4497378	2			
serX	1097463-1099253	1	*serX*	EG30097	Serine tRNA
proL	2285653-2287007	1	*proL*	EG30067	Proline tRNA
nagB	702668-703846	1	*nagB*	EG10633	Glucosamine-6-phosphate deaminase
sseA,ryfA,sseB	2652072-2656153	1	*sseA*	EG11600	3-mercaptopyruvate:cyanide sulfurtransferase
ypaB	2343041-2345271	2	*ypaB*	G0-10596	Hypothetical protein
ydfG	1626675-1628844	1	*ydfG*	EG12345	3-hydroxy acid dehydrogenase.
yiiQ,yiiR,yiiS, uspD	4111180-4114078	1	none[c]		UspD (UV resistance); YiiQ, YiiR,YiiS (hypothetical proteins)

S1 Fig. Overview of the multicopy suppressors of the Δ*tig* Δ*dnaKJ* mutant, related to Figure 1. (**A**) Overview of the genes capable of suppressing as single gene the temperature sensitive phenotype of MG1655 Δ*tig* Δ*dnaKJ* mutant. The genes involved in CM studied in this work are shown in blue color. Note that *csrB* was also included in this work, although not isolated in the genetic selection, as described in the main text. Asterisk indicates that the suppressor genes were described elsewhere [1-4]. (**B**) Details about suppressors isolated in this work. [a] Genomic fragments (nucleotide position on the MG1655 genome) of the pMPMA2-based multicopy suppressors isolated on the basis of growth complementation of MG1655 Δ*tig* Δ*dnaKJ* mutants (materials and methods); [b] When multiple genes were initially present on the plasmid suppressor, the gene responsible for the suppression at 35°C was identified following sub-cloning into the IPTG-inducible plasmid pSE380ΔNcoI; [c] indicates that overexpression of each gene separately did not recue bacterial growth, suggesting that expression of two or more genes might be responsible for the suppression observed. Only genes that are capable of suppressing as single gene were considered *bona fide* suppressors. The CM genes related to this study are underlined in blue color.

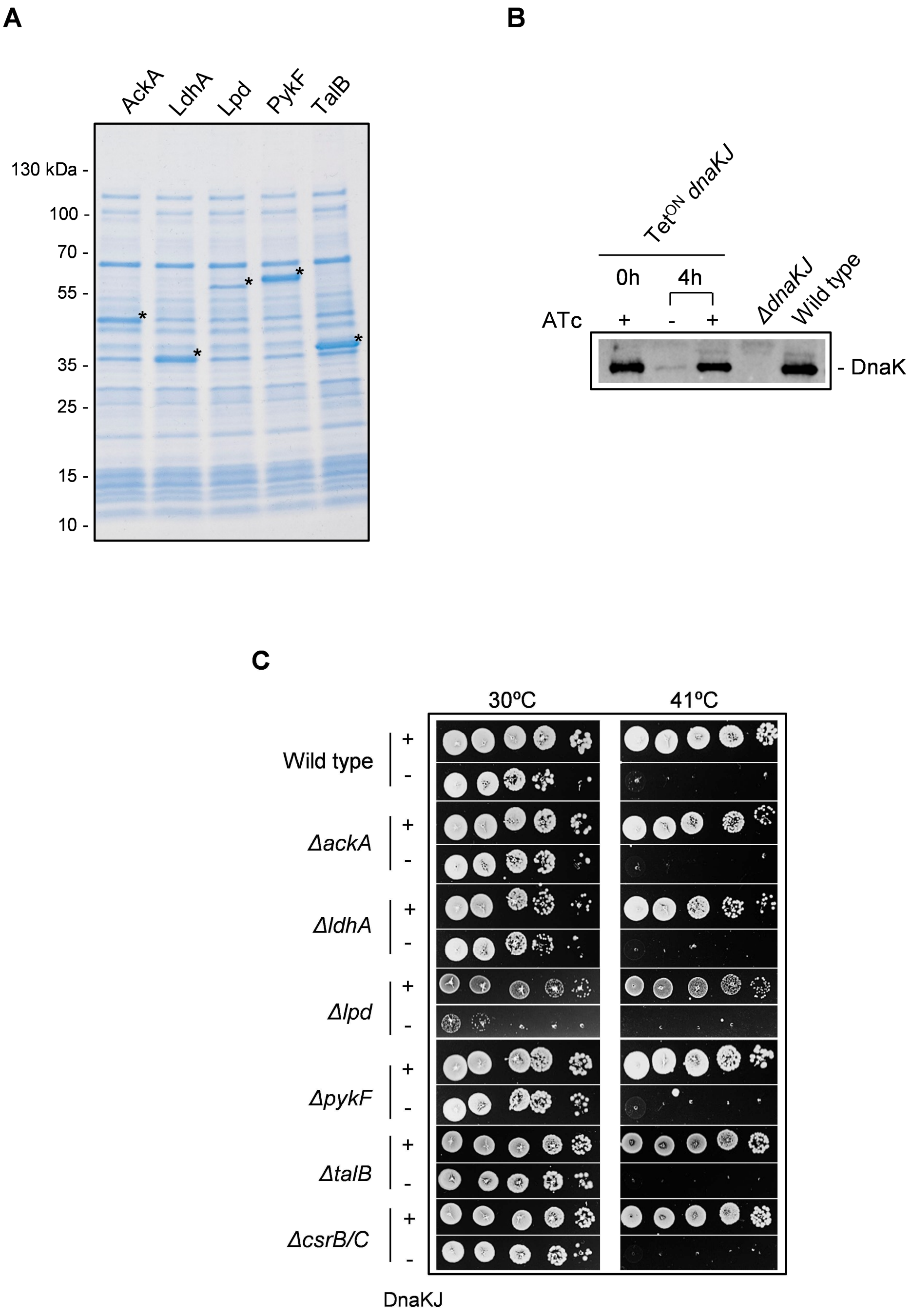

S2 Fig. Genetic interactions. (**A**) Overexpression of AckA, LdhA, Lpd, PykF and TalB in the absence of DnaK and TF, related to Fig 1. *E. coli* MG1655 Δ*tig* Δ*dnaKJ* transformed with pSE-AckA, pSE-LdhA, pSE-Lpd, pSE-PykF and pSE-TalB was grown overnight in LB medium supplemented with ampicillin (50µg/ml) glucose 0.4% at 22°C. Culture both were diluted 1/50 in fresh medium and cells were grown at 22°C until OD_{600} 0.3 at which time IPTG was added. After 3 hours, whole cell extracts were prepared and proteins were

separated on SDS-PAGE prior to be stained with Coomassie Blue. (**B**) DnaKJ expression under the control of the PTet^{ON} promoter. The Tet^{ON} *dnaKJ* is an MG1655 derivative in which the endogenous *dnaKJ* promoter is replaced by the tetracycline promoter P_{tet}, together with the upstream *terR* repressor [5]. In this case, expression of DnaK is dependent on the presence of anhydrotetracycline (100µg/l). Overnight cultures of *E. coli* K-12 MG1655 PTet^{ON} *dnaKJ* was grown in LB supplemented with kanamycin (50µg/ml) and anhydrotetracycline at 30°C. Cells were then washed, diluted 1/50 in LB and grown during 4 hours with or without anhydrotetracycline at 30°C. DnaK were revealed by western blot using anti-DnaK antibody. (**C**) Growth of double *dnaK* and CM gene mutants. Construction of the double Δ*ackA*Δ*dnaK,* Δ*ldhA*Δ*dnaK,* Δ*lpd*Δ*dnaK,* Δ*pykF*Δ*dnaK,* Δ*talB*Δ*dnaK,* and triple Δ*csrB*Δ*csrC*Δ*dnaK* mutants was performed by introducing the Δ*dnaK*::Cm^R *thr*::Tn*10* mutant allele into MG1655 Δ*ackA,* Δ*lpd,* Δ*ldhA,* Δ*pykF,* Δ*talB or* Δ*csrB*Δ*csrC* mutants. *E. coli* K-12 MG1655 wild type, Δ*dnaKJ,* Δ*ackA,* Δ*ackA* Δ*dnaKJ,* Δ*ldhA,* Δ*ldhA*Δ*dnaKJ,* Δ*pykF,* Δ*pykF*Δ*dnaKJ,* Δ*talB,* Δ*talB*Δ*dnaKJ,* Δ*csrB/C,* Δ*csrB/C*Δ*dnaKJ,* Δ*lpd and* Δ*lpd*Δ*dnaKJ* strains were grown at 30 °C, serially diluted 10-fold, and spotted on LB agar plates. Plates were incubated for 1 day at temperatures mentioned on the figure.

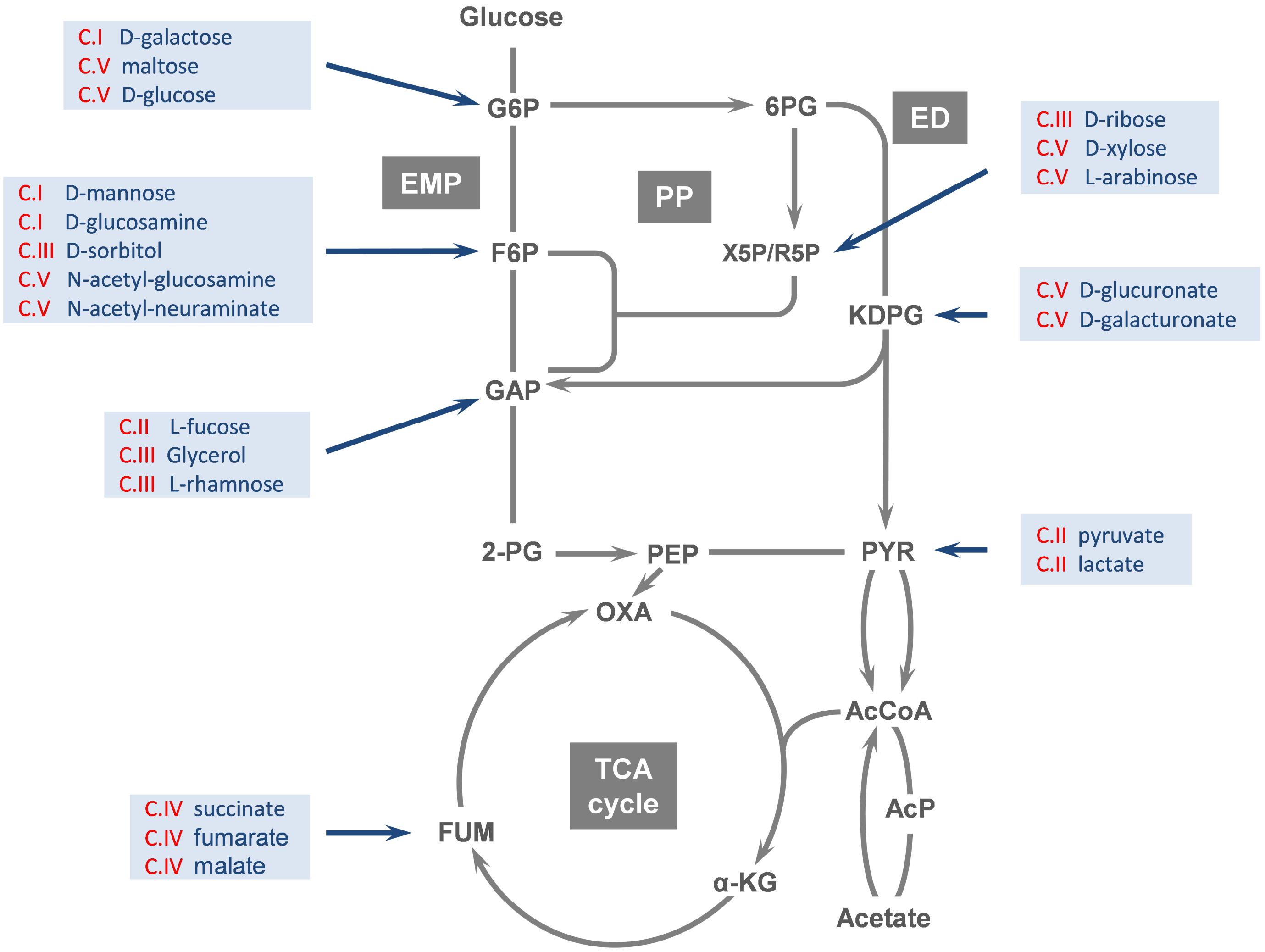

S3 Fig. Entry points of the different carbon sources used. The metabolic network includes the Embden–Meyerhof–Parnas (EMP) pathway, the Pentose Phosphate (PP) pathway, the Entner-Doudoroff (ED) pathway and the Tricarboxylic Acid (TCA) cycle. The different classes of carbon sources defined in Fig 2 are indicated in front of each carbon source (from C.I to C.IV). Utilization of maltose, D-glucose and D-galactose, yields to glucose-6-phosphate (G6P), the first metabolic intermediate of EMP pathway; utilization of D-glucuronate or D-galacturonate converges towards the ED pathway at level 2-keto-3-deoxy-6-phospho-D-gluconate (KDPG); utilization of D-sorbitol, D-mannose, D-glucosamine, N-acetyl-glucosamine and the N-acetyl-neuraminate converges towards the fructose-6-phosphate

(F6P), the second hexose-P of the EMP pathway; glycerol is metabolized in dihydroxyacetone phosphate, one of the two triose-phosphates generated in the EMP pathway; utilization of L-fucose and L-rhamnose also converges towards the formation of glyceraldehyde-3-phosphate (GAP) while being also converted into lactaldehyde; L-lactate and pyruvate enter at the level of the pyruvate node; the pentoses, D-xylose, L-arabinose and D-ribose yield intermediates of the PP pathway after isomerization and phosphorylation steps; succinate, fumarate and malate enter the CM at the level of the TCA cycle . Other abbreviations: 6-phosphogluconate (6PG), ribose-5-phosphate (R5P), xylulose-5-phosphate (X5P), 2-phosphoglycerate (2PG), phosphoenolpyruvate (PEP), pyruvate (PYR), acetyl-CoA (AcCoA), acetyl-phosphate (AcP) alpha-ketoglutarate (a-KG), oxaloacetate (OXA).

A

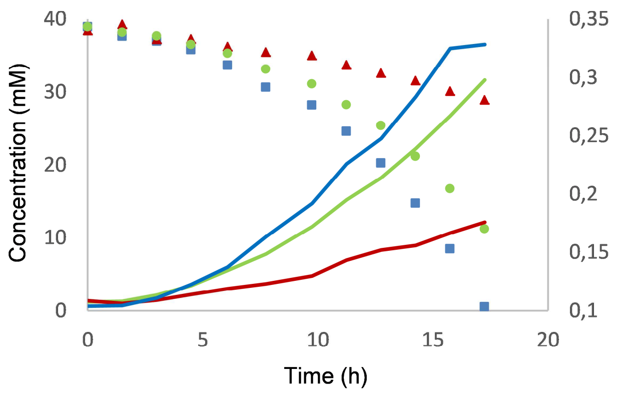

	Wild type	*ΔdnaKJ*	*rpoH*$^{(I54N)}$
μ (h-1)	0,07 ± 0,1	0,03 ± 0,1	0,08 ± 0,1
q_S (mmol.g_{DW}^{-1}.h^{-1})	17,8 ± 1,5	11,1 ± 1,0	26,9 ± 1,9

B

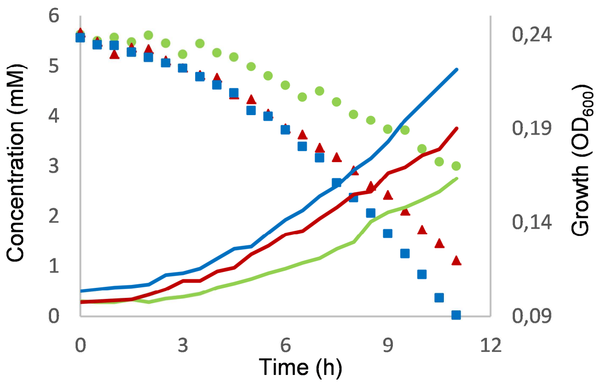

	Wild type	*ΔdnaKJ*	*rpoH*$^{(I54N)}$
μ (h-1)	0,04 ± 0,1	0,07 ± 0,1	0,07 ± 0,1
q_S (mmol.g_{DW}^{-1}.h^{-1})	4,6 ± 0,4	8,6 ± 1,4	9,3 ± 1 ,3

C

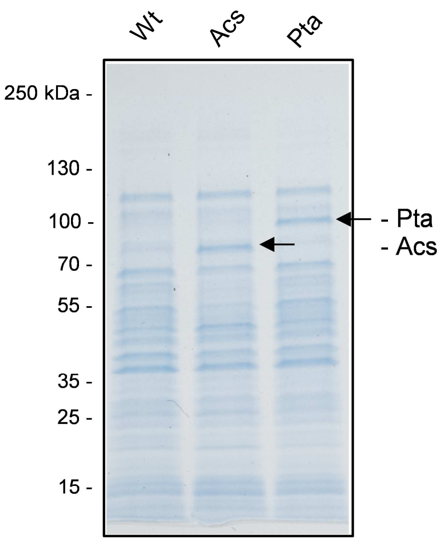

D

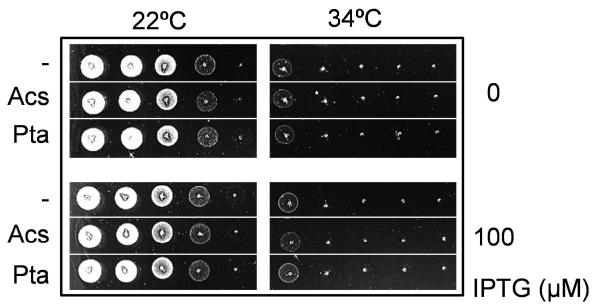

S4 Fig. DnaK differentially affects the utilization of acetate as carbon source. The absence of DnaK slows down the utilization of acetate at high concentration, the heat-shock response increases acetate utilization at low concentration. AckA is the only protein involved in the acetate utilization to rescue bacterial growth in the absence of DnaKJ and TF when overexpressed (see Fig 1). Growth profile of *E. coli* MG1655 wild type (green), Δ*dnaKJ* (red) and *rpoH*$^{(I54N)}$ (blue) on 40mM (**A**) or 6 mM (**B**) of acetate. (**C**) Overnight cultures of *E. coli* MG1655 Δ*tig* Δ*dnaKJ* transformed with pSE-Acs and pSE-Pta were grown in LB

supplemented with ampicillin (50µg/ml) and glucose 0.4% at 22°C. Culture broth were then diluted 1/50 with fresh LB medium and cells were grown at 22°C until OD_{600} 0.3 at which time IPTG 500µM was added. Whole cell extracts were prepared and proteins were separated on SDS-PAGE prior to be stained with Coomassie Blue. (**D**) Fresh transformants of strain MG1655 *tig*::Cm^R *dnaKJ*::Kan^R containing the plasmid pSE380NcoI parental vector, pSE-Acs and pSE-Pta were grown at 22 °C, serially diluted 10-fold, and spotted on LB ampicillin agar plates with or without IPTG inducer. Plates were incubated for 1 day at 34 °C or 2 days at 22 °C.

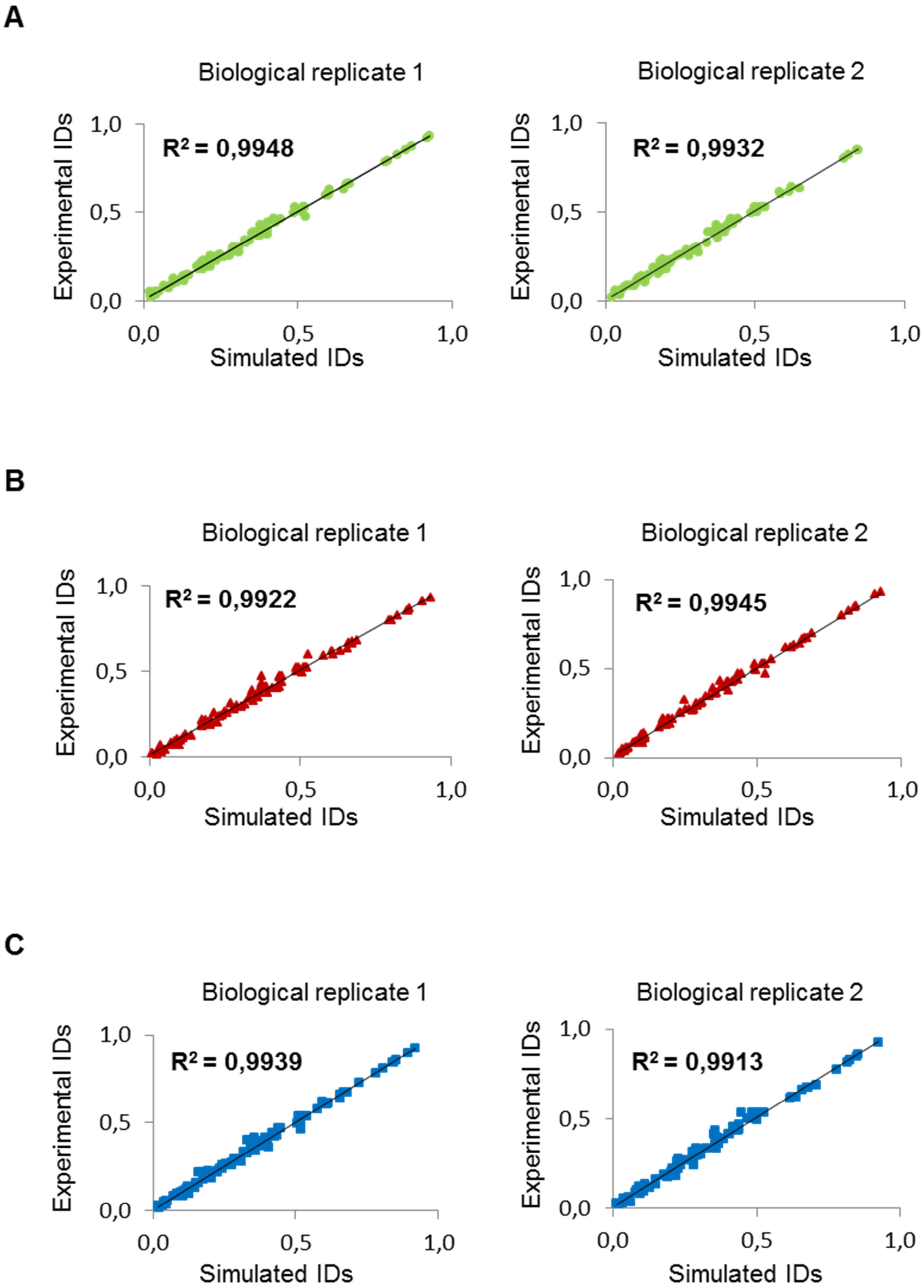

S5 Fig. Correlation between measured and simulated Isotopic Data for Wild type (A), Δ*dnaKJ* (B) and *rpoH*$^{(I54N)}$ **(C).**

REFERENCES

1 Bruel, N. *et al.* Hsp33 Controls Elongation Factor-Tu Stability and Allows Escherichia coli Growth in the Absence of the Major DnaK and Trigger Factor Chaperones. *J Biol Chem* **287**, 44435-44446, doi:10.1074/jbc.M112.418525 (2012).

2 Ullers, R. S. *et al.* SecB is a bona fide generalized chaperone in Escherichia coli. *Proc.Natl.Acad.Sci.U.S.A* **101**, 7583-7588 (2004).

3 Vorderwulbecke, S. *et al.* Low temperature or GroEL/ES overproduction permits growth of Escherichia coli cells lacking trigger factor and DnaK. *FEBS Lett.* **559**, 181-187 (2004).

4 Genevaux, P. *et al.* In vivo analysis of the overlapping functions of DnaK and trigger factor. *EMBO Rep.* **5**, 195-200 (2004).

5 Perrody, E. *et al.* A bacteriophage-encoded J-domain protein interacts with the DnaK/Hsp70 chaperone and stabilizes the heat-shock factor sigma32 of Escherichia coli. *PLoS Genet* **8**, e1003037, doi:10.1371/journal.pgen.1003037 (2012).
